# The Use of Bulk Fill Resin-Based Composite in the Sealing of Cavity with Margins in Radicular Cementum

**DOI:** 10.1055/s-0041-1731834

**Published:** 2021-09-10

**Authors:** Puleio Francesco, Cervino Gabriele, Luca Fiorillo, Miragliotta Giuseppe, Squillacioti Antonella, Bruno Giancarlo, Pinizzotto Mirta, João Paulo Mendes Tribst, Roberto Lo Giudice

**Affiliations:** 1 Department of Biomedical and Dental Sciences and Morphofunctional Imaging, Messina University, Messina, Italy; 2 Multidisciplinary Department of Medical-Surgical and Odontostomatological Specialties, University of Campania “Luigi Vanvitelli,” Naples, Italy; 3 Department of Dental Materials and Prosthodontics, Institute of Science and Technology, São Paulo State University, São José dos Campos, Brazil; 4 Department of Clinical and Experimental Medicine, Messina University, Messina, Italy

**Keywords:** bulk fill resin, composite, conservative dentistry, radicular cementum, sealing

## Abstract

The aim of this systematic review was to evaluate if the newly introduced bulk fill resin-based composite provides a better marginal sealing in cavity preparations with margins in dental cementum. The population investigation comparison outcome (PICO) framework was: in cavity preparation with margins in dental cementum of human extracted teeth, do bulk fill resin base composites provide a better marginal sealing than non-bulk fill resin-based composites? We performed our research on April 21, 2020. Two authors independently evaluated the abstract and titles for eligibility criteria. Two authors independently extracted the data and assessed the risk of bias in single studies. After the initial screening of 400 abstract and titles, the full text of the articles, that could meet the eligibility criteria, were obtained via the university library. A total of 36 full-text articles were evaluated; 11 articles were finally eligible for the review. Eight studies showed statistically differences, but not significant, in the marginal sealing between bulk fill and nonbulk fill resin-based composite ( *p* > 0.05). One study showed statistically significant differences: SonicFill and Grandio showed better marginal sealing than GrandioSo and SDR(r) (Sirona Dentsply, New York, United States) and the latter two showed better marginal sealing than Filtek Supreme ( *p* < 0.05). One study showed statistically significant less marginal gap of SDR than Filtek Bulk Fill ( *p* = 0.0015) and Filtek Supreme ( *p* < 0.0001). One study showed SDR to have a significantly higher microleakage than the other materials tested ( *p* < 0.05). Based on our current literature review, there are not enough data to establish if bulk fill resin base composite provides a better or a worse marginal sealing at cementum margins.

## Introduction

### Rationale

With the increase in the average age and with the general improvement in the oral health conditions of the population, there is more and more often a greater presence of dental elements in the oral cavity of older patients than in previous times. 1 The prevalence of root surface exposures correlates positively with the age of the patient: consequently, the prevalence of carious involvement of the root surface increases. 2 Root cementum is a surface with a reactivity 3 greater than that of enamel and this makes it more susceptible to the action of endogenous metalloproteases as well as bacterial ones. 4

Microleakage is defined as the passage of bacteria and their toxins through the margins of the restoration and the tooth surface of the cavity preparation. 5 The anatomical basis of this phenomenon is the marginal gap between the restoration and the dental tissues; the clinical implications could be postoperative sensitivity, dentinal sensitivity, and development of secondary caries. 6 Hydrolytic degradation of the adhesive bond, which can occur both on the adhesive component and on the collagen of the dental tissue, happens more frequently when the margins of the restoration are not placed in enamel. 7

As highlighted by Lo Giudice et al, 8 a restoration that has a cement margin represents challenge for adhesive dentistry techniques: in fact, the higher percentage of organic material (23%) of the root cementum, compared with enamel (1–2%), makes cementum a substrate that exhibits weaker and less predictable adhesion parameters. Furthermore, it must be emphasized that the presence of failure and marginal fractures is not eliminated by the use of new adhesives with higher adhesion values. 9 10 Adhesion to cement/dentin is in fact the weak point of the adhesive restoration due to several factors: hydrolysis of the adhesive layer, inadequate infiltration of the adhesive into the substrate, and incomplete evaporation of the solvent. Some of these drawbacks can be modified by varying the type of adhesive strategy 11 : for example, the use of functional monomers inside the adhesive makes possible to obtain an adhesive layer through the phenomenon of “nano layering” 12 preventing hydrolytic degradation of the adhesive layer. 13 Evidence also suggests that a total etch approach produces less marginal discoloration *in vivo* , without improving postoperative sensitivity, 14 but in conclusion neither a total etch approach nor a self-etch approach can guarantee the development of a hybrid layer without porosity. 15

The marginal gap is directly correlated to the shrinkage stress of the material and its elastic modulus. The elastic modulus is a characteristic of the material, while the shrinkage stress is related both to the material and to other factors (for example the cavity configuration). 16 The gold standard materials for restorative dentistry are resin-based composites due to their characteristics. 17 These materials also find application in the cervical region of the dental element where they show better *in viv* o performances than glass ionomer cements. 18

One of the major drawbacks of traditional composite materials is their polymerization shrinkage, which can also be considerable and can reach 3 to 7% of the initial mass, 19 20 21 22 contributing to the formation of marginal gap.

Traditional composites must be deposited using the incremental technique both to reduce the effects of polymerization shrinkage and to promote complete polymerization of the material. 23 24 25 26 It has also been shown that the use of the composite with the bulk technique produces a high cusp deflection. 27 28

The need for a material with low polymerization shrinkage has led to the development of a several resin-based composite materials (siloranes, ormocers, nano-filled composites) that exhibit lower polymerization shrinkage than conventional composites. However, they still need to be deposited in maximum increments of ~2 mm due to their limited depth of polymerization 29 and some are also impractical due to the need for a specific adhesive system. 30 31 A further improvement in the technology has led to the development of materials that have both a reduced polymerization shrinkage and an increased depth of cure (DOC). The combination of these two characteristics allows the material to be deposited in increments greater than 2 mm of the nonbulk composite.

The optimization of the DOC is achieved through different strategies:

- An increase in translucency 32 33 typical of all bulk composites, with the exception of SonicFill (Kerr);
- A modulation of the photo-polymerization that is obtained, in addition to the presence of camphorquinone and tertiary amines, by specific modulators. Tetric EvoCeram Bulk Fill (Ivoclar Vivadent) contains an additional photo-initiator: Ivocerin (derivative of dibenzoyl germanium). 34
- By new functional monomers: many bulk composites contain modified Uretandimetacrylate (UDMA) with photo-active groups that would act as modulators of photo-polymerization. 35

In 2010, the first resin-based composite was developed that could be deposited in increments of up to 4 mm. 36 This new class of materials is collectively referred to as “bulk fill resin-based composites.” These composites are heterogeneous in composition and commercial presentation therefore a satisfactory classification is difficult, if not impossible. However, classifying bulk composites according to viscosity, we can divide bulk composites into three classes (see Tables 1 and 2 ).

**Table 1:** Main studies results (Part I)

	Juloski et al 2013 38	Poggio et al 2013 39	Al-Harbi et al 2016 43	de Assis et al2016 44	Scotti et al 2014 40	García Marí et al 2019 48	Haak et al 2018 46
Numerosity	50 cavities	100 cavities	91 cavities	40 cavities	48 cavities	80 cavities	64 interproximal box (128 cavities MOD)
Cavity dimensions	7(x2og) (md)4(ov)mm	4(ov)mm, gingival wall 2mm below CEJ.	4mm (ov) × 1.5mm (md)	5x2x2mm: n 20 5x4x2mm:n 20	2 mm × 2 mm × 2 mm	4mm (og)x4mm (ov), 2mm (md)	MOD designed: 4mm buccolingual, 4mm occlusoapical Box interproximal: 2mm mesiodistal, 5mm buccolingual, 1–2mm below la CEJ
Material tested	1-G-aenial Bond/G-aenial Flo bulk (GB/GF) 1-GB/G-aenial Universal Flo bulk (GUF) 3-GB/GC Kalore bulk fill (GK) 4-DeTrey Conditioner 36 etching gel (EG)/XP Bond(XB)/ SureFill SDR flow (SDR) 5- Prime (PSA) and bond (BSA) silorane Adhesive System/Filtek Silorane (FS)	Filtek TM Supreme 1 XTE Flowable 2 vs. SDR 3 vs. Sonic Fill 4 vs. Grandio 5	1-Tetric Ceram HB (TC)(SE/TE) vs. 2-Tetric EvoFlow (EF) (SE/TE) vs. 3-Smart Dentin Replacement (SD) (SE/TE) vs. 4-SonicFill (SF) bulk(SE/TE) vs. 5-Tetric N-Ceram Bulk Fill (TN), bulk (SE/TE) vs. 6-Tetric EvoCeram Bulk Fill (TE). (SE/TE) vs. 7 Filtek P90 Low Shrink Posterior Restorative (P9)(SE)	“Surefil SDR Flow (Dentsply) vs. TPH3 Spectrum (Dentsply)”	Venus Diamond(1A)(1B) vs. Venus diamond flow(2A)(2B) vs. Surefil SDR flow(3A)(3B)	FiltekTM Bulk Fill A2(Group 1) vs. Filtek Supreme XTE A2B (Group 2)	SonicFill (SF) OFL/X, vs. Tetric EvoCeram Bulk fill (TEC) OFL/X, vs. x-tra fil (XF) OFL/X vs. Premise (P) OFL/X
Aging procedures	Storage in distilled water 37°C per 24 h	Thermocycling	Thermocycling and occlusal load	Storage in distilled water 37°C	No one	Thermocycling	Storage in distilled water 37°C for 24 h or for 180 d
Evaluation of marginal integrity	Immersion in silver nitrate, then in development solution, then analyzed sections at 2x magnification	Immersion in 0.5% basic fuchsin solution	Analysis of the copies in epoxy resin. SEM 200X	Adhesive interface observation of the epoxy resin copies at 400X	Analysis before and after artificial aging. Methylene blue 1:10	Immersion in 0.5% basic fuchsin solution	Analysis of the copies in epoxy resin SEM 200X
Results	Median score (25–75%) Group1: median score 0 (0–0), Group2: median score 0 (0–1) Group3: median score 0 (0–1) Group4: median score 2 (2–2) Group5: median score 0 (0–0)	Group 4 e 5”score 0” significantly prevalent, Group 2 e 3 “score1”significantly prevalent, Group 1 prevalenza significativa di “score 2”significantly prevalent.	Perfect margin percentage value (PMP%): TC/SE 70.0 ±21.5; TC/TE 82.3 ±22.1; TC+EF/SE 88.7 ±19.3; TC+EF/TE 88.3 ±20.1; TC+SD/SE 79.3 ±25.9; TC+SD/TE88.0 ±20.8; SF/SE 90.1 ± 17.2; SF/TE 93.3 ±17.8; TN/SE 86 ± 24.0; TN/TE 81.4 ± 23.5; TE/SE 91.0 ±23.8; TE/TE 76.9 ±29.3; P9/SE89.4 ±28.0	Marginal integrity (%). Conservative/bulk: 95.9 ± 2.6; Extended/ bulk 94.4 ± 6.7; Conservative/Incremental 93.5 ± 5.8; Extended/Incremental 90.9 ± 8.4.	Marginal microleakage percentage before (A) and after aging(B): 1A 45.69, 2A 32.32, 3A 30.45. 1B 53.10, 2B 40.04, 2C 37.18.	Microleakage percentage. Group1: “score0” 10%, “score1” 25%, “score2” 40%, “score3” 25%. Group2 “score0” 5%, “score1” 50%, “score2” 35%, “score3” 10%.	Marginal gap formation (%). SF/OFL-24h 1.5 ± 2.4, SF/OFL-6mesi 5.4 ± 4.7; SF/X-24h 21.7 ± 22.5, SF/X-6mesi 42.8 ± 31.4; TEC/OFL-24h 8.9 ± 9.1, TEC/OFL-6mesi 20.0 ± 22.0; TEC/X-24h 5.5 ± 6.4, TEC/X-6mesi 12.1 ± 12.4; XF/OFL-24h 2.2 ± 3.8, XF/OFL-6mesi 3.9 ± 3.8; XF/X-24h 17.2 ± 24.8; XF/X-6mesi 23.1 ± 15.1; P/OFL -24h 6.2 ± 9.8, P/OFL-6mesi 9.6 ± 16.4, P/X-24h 5.1 ± 2.9, P/X-6mesi 26.3 ± 16.5
Statistical analysis	*p* <0.05	*p* <0.05	*p* = 0.848	*p* = 0.77	*p* <0.204	*p* = 0.468	*p* >0.05

**Table 2:** Main studies results (Part II)

		Webber et al 2014 41			Kalmowicz et al 2015 42		Behery et al 2018 45			Peutzfeldt et al 2018 47	
Numerosity		20 cavities			40 cavities		80 cavities			78 cavities	
Cavity dimensions		MOD cavity proximal margin 1mm above CEJ, Distal margin 1mm below CEJ. 3mm occlusopulpal 3mm (ov).			3mm buccolingual × 1.5mm axial depth × 4mm height		3mm (ov) × 1.3mm (md)			4mm (ov), 6mm (oc), 2mm (md)	
Material tested		SureFil SDR (Dentsply) 1 vs. TPH3 composite (Dentsply) 2			SonicFill (B) vs. Herculite Ultra (D)		Tetric EvoCeram Bulk Fill(1)24h/6mesi, vs. X-tra Fil (2) 24h/6mesi vs. QuiXX (3) 24h/6mesi vs. TPH Spectra HV (Control) (4) 24h/6mesi			Filtek SupremeXTE(1) Filtek Bulk Fill(2) SDR(3)	
Aging procedures		Thermocycling			No one and thermocycling		Storage for 24h and 6 mo			Storage in water for 24h, then mechanical brushing and thermocycling	
Evaluation of marginal integrity		0.5% basic fuchsin immersion			In a solution of 1% methylene blue		2% procion red solution (Imperial Chemical Industries, London, England)			Analysis before and after artificial aging Analysis of the copies in epoxy resin SEM 200X	
Results		Group 1: 10 “score 2”; Group 2: 2 “score 0,” 1 “score1,” 5 “score 2,” 2 “score3,”			Group B: median score 2,487 ± 1,091; Group D: median score 2,775 ± 0,795		Gingival score microleakage. Group1/24h 0.30 ± 0.48, Group 1/mo 0.90 ± 1.29; Group2 /24h 0.50 ± 0.71, Group2/6 mo 1.10 ± 1.10; Group 3/24h 0.40 ± 0.70, Gruppo 3/6mo 0.90 ± 0.88; Group 4/24h 0.40 ± 0.70, Group 4/6mo1.00 ± 1.05.			Marginal gap formation (%). Group1(SM) baseline 2.9 ± 4.8, finale 4.1 ± 6.2, (T) baseline 4.6 ± 8.5, final 4.6 ± 8.9. Group2 (SM) baseline 7.9 ± 27.7, final 9.1 ± 26.9, (T) baseline 7.9 ± 27.5, final 19.1 ± 28.6. Group3(SM) baseline 0 ± 0, final 7.0 ± 19.6, (T) baseline 0 ± 0, final 0 ± 0	
Statistical analysis		*p* = 0.195			*p* = 0.0586		Difference at 24 h *p* = 0.945, Difference at 6 mo, *p* = 0.928.			Group3 less gap than Group 1 ( *p* < 0.0001), Group3 less gap than Group2 ( *p* = 0.0015). Group 1 e group 2 non no difference ( *p* = 0.4919)	
Cavity dimensions	7(og)x2(md)4(ov)mm		4(ov)mm, gingival wall 2mm below CEJ.	4mm (ov) × 1.5mm (md)		5x2x2mm: n 20 5x4x2mm:n 20		2 mm × 2 mm × 2 mm	4mm (og)x4mm (ov), 2mm (md)		MOD designed: 4mm buccolingual, 4mm occlusoapical, Box interproximal: 2mm mesiodistal, 5mm buccolingual, 1–2mm below la CEJ
Material tested	1-G-aenial Bond/G-aenial Flo bulk (GB/GF) 1-GB/G-aenial Universal Flo bulk (GUF) 3-GB/GC Kalore bulk fill (GK) 4-DeTrey Conditioner 36 etching gel (EG)/XP Bond(XB)/ SureFill SDR flow (SDR) 5- Prime (PSA) and bond (BSA) silorane Adhesive System/Filtek Silorane (FS)		Filtek TM Supreme 1 XTE Flowable 2 vs. SDR 3 vs. Sonic Fill 4 vs. Grandio 5	1-Tetric Ceram HB (TC)(SE/TE) vs. 2-Tetric EvoFlow (EF) (SE/TE) vs. 3-Smart Dentin Replacement (SD) (SE/TE) vs. 4-SonicFill (SF) bulk(SE/TE) vs. 5-Tetric N-Ceram Bulk Fill (TN), bulk (SE/TE) vs. 6-Tetric EvoCeram Bulk Fill (TE). (SE/TE) vs. 7 Filtek P90 Low Shrink Posterior Restorative (P9)(SE)		“Surefil SDR Flow (Dentsply) vs. TPH3 Spectrum (Dentsply)”		Venus Diamond(1A)(1B) vs. Venus diamond flow(2A)(2B) vs. Surefil SDR flow(3A)(3B)	FiltekTM Bulk Fill A2(gruppo1) vs. Filtek Supreme XTE A2B (gruppo2)		SonicFill (SF) OFL/X vs. Tetric EvoCeram Bulk fill (TEC) OFL/X vs. x-tra fil (XF) OFL/X vs. Premise (P) OFL/X
Aging procedures	Storage in distilled water 37°C per 24h		Thermocycling	Thermocycling and occlusal load		Storage in distilled water 37°C		No one	Thermocycling		Storage in distilled water 37°C for 24h or for 180 d
Evaluation of marginal integrity	Immersion in silver nitrate, then in development solution, then analyzed sections at 2x magnification		Immersion in 0.5% basic fuchsin solution	Analysis of the copies in epoxy resin. SEM 200X		Adhesive interface observation of the epoxy resin copies at 400X		Analysis before and after artificial aging. Methylene blue 1:10	Immersion in 0.5% basic fuchsin solution		Analysis of the copies in epoxy resin. SEM 200X
Results	Median score (25–75%) Group1: median score 0 (0–0), Group2: median score 0 (0–1) Group3: median score 0 (0–1) Group4: median score 2 (2–2) Group5: median score 0 (0–0)		Group 4 e 5”score 0” significantly prevalent, Group 2 e 3 “score1”significantly prevalent, Group 1 prevalenza significativa di “score 2”significantly prevalent.	Perfect margin percentage value (PMP%): TC/SE 70.0 ±21.5; TC/TE 82.3 ±22.1; TC+EF/SE 88.7 ±19.3; TC+EF/TE 88.3 ±20.1; TC+SD/SE 79.3 ±25.9; TC+SD/TE88.0 ±20.8; SF/SE 90.1 ± 17.2; SF/TE 93.3 ±17.8; TN/SE 86 ± 24.0; TN/TE 81.4 ± 23.5; TE/SE 91.0 ±23.8; TE/TE 76.9 ±29.3; P9/SE89.4 ±28.0		Marginal integrity (%). Conservative/bulk: 95.9 ± 2.6; Extended/ bulk 94.4 ± 6.7; Conservative/Incremental 93.5 ± 5.8; Extended/Incremental 90.9 ± 8.4		Marginal microleakage percentage before (A) and after aging(B): 1A 45.69, 2A 32.32, 3A 30.45. 1B 53.10, 2B 40.04, 2C 37.18	Microleakage percentage. Group1: “score0” 10%, “score1” 25%, “score2” 40%, “score3” 25%. Group2 “score0” 5%, “score1” 50%, “score2” 35%, “score3” 10%.		Marginal gap formation (%). SF/OFL-24h 1.5 ± 2.4, SF/OFL-6mesi 5.4 ± 4.7; SF/X-24h 21.7 ± 22.5, SF/X-6mesi 42.8 ± 31.4; TEC/OFL-24h 8.9 ± 9.1, TEC/OFL-6mesi 20.0 ± 22.0; TEC/X-24h 5.5 ± 6.4, TEC/X-6mesi 12.1 ± 12.4; XF/OFL-24h 2.2 ± 3.8, XF/OFL-6mesi 3.9 ± 3.8; XF/X-24h 17.2 ± 24.8; XF/X-6mesi 23.1 ± 15.1; P/OFL -24h 6.2 ± 9.8, P/OFL-6mesi 9.6 ± 16.4, P/X-24h 5.1 ± 2.9, P/X-6mesi 26.3 ± 16.5
Statistical analysis	*p* <0.05		*p* <0.05	*p* = 0.848		*p* = 0.77		*p* <0.204	*p* = 0.468		*p* >0.05

- Bulk-fill resin based composite (RBC) with low viscosity or “base,” used as the base of the restoration that must be covered by a layer of no-bulk composite (according to the manufacturer's instructions).
- Bulk-fill high viscosity or “full body” that can be used throughout the restoration, but may sometimes require a nonbulk composite cap (according to the manufacturer's instructions).

### Bulk-Fill RBC That Require Sonic Activation

The practical consequences are considerable. The positioning of a restoration with a cement margin represents a challenge for the clinician associated with the problem of time: the cervical area, in fact, is difficult to control and to access; moreover, it is difficult to maintain adequate isolation for a relatively long period. Moreover, it is shown that the contamination of the cavity preparation with a hemostatic agent significantly reduces the marginal seal on cement, interfering with the adhesive procedures. 37

Therefore, the possibility of using a material that has optimized physical characteristics and also allows shorter processing times is fascinating.

### Objectives

Our aim is to answer the following question according to the PICOS scheme: in cavity preparation with margins in dental cementum of human extracted teeth, do bulk fill resin base composites provide a better marginal sealing than non-bulk fill resin-based composites?

## Methods

### Eligibility Criteria

The search strategy, performed using the PubMed controlled vocabulary and free terms, was defined on the basis of the following elements of the PICO question:

Population (P): Cavity preparations with at least one margin in root cementum of human teeth.

Intervention (I): restoration made with a bulk fill composite.

Comparison (C): restoration made with a nonbulk composite (resin-based composite).

Outcome (O): marginal integrity and/or microleakage

Study design (S): *in vitro* studies.

The eligibility criteria are: *in vitro* studies, published in the last 10 years (given the date of introduction of the material on the market) and written in English, it was decided to include studies on human teeth and *in vitro* studies to have a standardization of the cavity, which would not be possible to obtain in an *in vivo* study.

Were included studies that explicitly described that a cavity margin of the preparation was in the root cement. Finally, comparative studies between a bulk fill composite and a nonbulk composite were chosen.

### Information Sources

To identify the literature of our interest, a search was performed on PubMed, Scopus, Google Scholar, Semantic Scholar, and on gray literature via OpenGrey.eu.

### Search

In the construction of this research, we wanted to combine three fundamental concepts: the anatomical site, the concept of microinfiltration, and the material (Fig. 1).

On April 21, 2020, the following searches were performed:

### Search on PUBMED

# 1. “dental cementum” OR “root caries” OR “tooth root” OR dentin
# 2. “dental leakage” OR “dental marginal adaptation”
# 3. “filtek bulk fill” OR “SDR composite” OR “composite resins” OR “dental bonding” OR “dentin-bonding agents” OR “dental cement” OR “resin cements”

(“Tooth Root” [Mesh] OR “Dentin” [Mesh] OR “Dental Cementum” [Mesh]) AND (“Dental Leakage” [Mesh] OR “Dental Marginal Adaptation” [Mesh]) AND (“Filtek Bulk Fill” [Supplementary Concept] OR “Composite Resins” [Mesh] OR “SDR composite” [Supplementary Concept] OR “Dental Bonding” [Mesh] OR “Dentin-Bonding Agents” [Mesh] OR “Dental Cements” [Mesh] OR “Resin Cements “[Mesh])

### Search on Scopus

# 1 Cementum OR “root surface” OR “dental cementum” OR “tooth root” OR “cement enamel junction” OR “tooth cementum” OR “tooth cervix” OR “dental cementum” OR “dental cementum” OR “root caries”
# 2 Microleakage OR “dental leakage” OR “cervical microleakage” OR “dental restoration failure” OR “mineral interfaces” OR “marginal quality” OR “gap formation” OR “thooth hypersensitivity”
# 3 “Composite resins” OR “dental composites” OR “resin-based composite” OR “bulk fill” OR “resin composite” OR “bulk-fill” OR “composite resin” OR “filtek bulk fill” OR “composite resin” OR “SDR composite” OR “dental bonding” OR “dentin bonding agents” OR “dental cement” OR “resin cement”

Additional searches were performed on Google scholar and semantic Scholar using the terms “bulk fill” and “microleakage”

A further search, which did not exclusively include PubMed indexed literature, was performed by combining the term “bulk fill,” using the AND operator, from time to time to the terms II class, III class and V class to try to introduce the studies that referred to the design of the cavity rather than the concept of “root cement”

The results were limited to the last 10 years and to studies performed on the human species.

### Study Selection

Two reviewers independently assessed the titles and abstracts of all of the studies. Any disagreement regarding the eligibility of the included studies was resolved through discussion and consensus or by a third reviewer.

### Data Collection Process

The data collection strategy was defined on the basis of the characteristics of the PICOS model, to these characteristics were added explicitly through a predefined table. Data was collected based on the default table by the two reviewers.

### Data Items

The Tables 1 and 2 evaluate the general data (year, title, and author), the size of the sample (the number of cavities), the dimensions of the cavity, the materials tested, whether an artificial aging procedure has been performed, and the method used to evaluate the marginal integrity (immersion in dye and which dye or Scanning electron microscope evaluation of the epoxy replicas) and the results.

Within each publication, the data and statistical significance characteristics of the only interfaces between the restoration and the margin of the cavity preparation in root cement were collected.

### Risk of Bias in Individual Studies

To define the validity of the individual studies, the risk of bias was determined using the “Cochrane Collaboration's tool for assessing risk of bias in randomized trials” ( Tables 3 4 5 ).

**Table 3:** Risk of bias (Part I)

Item	Poggio et al 2013 39	Juloski et al 2013 38	Scotti et al 2014 40	Webber et al 2014 41	Kalmowicz et al 2015 42	Al-Harbi et Al 2016 43	de Assis et al 2016 44	Haak et al 2018 46
Adequate sequence generation	Unclear	Unclear	No	No	Unclear	No	No	Unclear
Allocation concealment	No	No	No	No	No	No	No	No
Blinding of participants and personnel	No	No	No	No	No	No	No	No
Blinding of outcome assessment	Yes	No	No	Yes	Yes	No	Yes	Yes
Incomplete outcome data addressed	No	No	No	No	No	No	No	No
Free of selective reporting	Yes	Yes	Yes	Yes	Yes	Yes	Yes	Yes
Free of other bias	Yes	Yes	No	Yes	No	Yes	Yes	Yes

**Table 4:** Risk of bias (Part II)

	Behery et al 2018 45	Peutzfeldt et al 2018 47	García Marí et al 2019 48
Adequate sequence generation?	No	No	No
Allocation concealment?	No	No	No
Blinding of participants and personnel?	No	No	No
Blinding of outcome assessment?	No	No	Yes
Incomplete outcome data addressed?	No	No	No
Free of selective reporting?	Yes	Yes	Yes
Free of other bias?	Yes	Yes	Yes

**Table 5:** Risk of bias (Part III)

Item	Poggio et al 2013 39	Juloski et al 2013 38	Scotti et al 2014 40	Webber et al 2014 41	Kalmowicz et al 2015 42	Al-Harbi et al 2016 43	de Assis et al 2016 44	Haak et al 2018 46	Behery et al 2018 45	Peutzfeldt et al 2018 47	García Marí et al 2019 48
Random sequence generation											
Allocation concealment											
Blinding of participants and personnel											
Blinding of outcome assessment											
Incomplete outcome data											
Selective reporting											
Other bias											

### Synthesis of Results

A narrative summary of the studies included in the review was made. It was not possible to carry out a meta-analysis due to the heterogeneity of the studies.

## Results

### Study Characteristics

The 11 selected studies were deemed eligible based on the predetermined PICOS criteria. Methods: selected studies are *in vitro* studies, in English language

Participants: The studies evaluate experimental cavity preparations in human teeth, which expressly present at least one root cement margin, have a total of 691 (Fig. 1).

Intervention: The intervention was the restoration with a bulk fill composite and the outcome evaluated was the degree of marginal adaptation.

Outcome: Data relating microinfiltration or marginal integrity were evaluated using different systems.

The summary data can be found in Tables 1 and 2 .

### Risk of Bias

The risk of bias was performed using the “Cochrane Collaboration's tool for assessing risk of bias in randomized trials” ( Tables 3 4 5 ).

### Results of Individual Studies

In the study of Juloski et al, 38 50 cavities were prepared, then the following materials were tested SureFil SDR flow versus Filtek Silorane versus G-ænial Flo bulk fill versus G-ænial Universal Flo bulk fill versus Kalore bulk fill, and the samples were stored for 24 hours at 37°C in distilled water, then immersed in silver nitrate, then in developer solution, and then sections were analyzed at 2x magnification.

The results show that SDR has greater microleakage than other materials.

In the study of Poggio et al, 39 100 cavities were prepared and then were restored with four different materials: Filtek TM Supreme XTE Flowable(1), SDR(2), Sonic Fill(3), and Grandio(4). The samples were subjected to thermocycling and then immersed in a 0.5% basic fuchsin solution. The results show that there is a difference according to the different composite used:

Sonic Fill and Grandio perform better than the, GrandioSo and SDR. Worst performance is seen with Filtek supreme.

In the study of Scotti et al, 40 48 cavities restored with three different materials: Venus Diamond (1A) (1B), Venus diamond flow (2A) (2B), and Surefil SDR flow (3A) (3B). The samples were evaluated before (A) and after artificial aging (B) by thermocycling. The results show that the influence of the material is not statistically significant on the microleakage.

In the study by Webber et al, 41 20 cavities were prepared, restored with SureFil SDR (Dentsply) 1 and TPH3 composite (Dentsply). 2 The samples were subjected to thermal cycling and immersed in basic fuchsin. The results show that there is no statistically significant difference in microinfiltration.

In the study Kalmowicz et al, 42 40 cavities SonicFill (B), Herculite Ultra (D) were considered. An analysis was performed before and after thermocycling by immersion in methylene blue and observation. There were no statistically significant differences in microinfiltration.

In the study of Al-Harbi et al, 43 91 cavities assigned to 13 restorative approaches were considered: in particular, the different materials were combined with a self-etch or total etch adhesive technique, with the exception of Filtek P90 applied only with self-etch technique: Tetric Ceram HB (TC) (SE / TE), TetricEvoFlow (EF) (SE / TE), Smart Dentin Replacement (SD) (SE / TE), SonicFill (SF) bulk (SE / TE), Tetric N-Ceram Bulk Fill (TN), bulk (SE / TE), Tetric EvoCeram Bulk Fill (TE) (SE / TE), Filtek P90 Low Shrink Posterior Restorative (P9) (SE). The specimens were subjected to thermal and occlusal cycling. SEM analysis of the copies in epoxy resin was performed. There is no statistically significant difference in marginal integrity.

In the study of de Assis et al, 44 40 cavities were considered, assigned to two different restorative protocols: Surefil SDR Flow (Dentsply) and TPH3 Spectrum (Dentsply). Samples were stored in distilled water at 37°C, and copies in SEM epoxy resin were observed. There is no statistically significant difference in marginal integrity between the materials tested.

In the study of Behery et al, 45 80 cavities were prepared for restoration with four different materials: Tetric EvoCeram Bulk Fill(1) 24 hours/6 months, X-traFil(2) 24 hours/6 months, QuiXX(3) 24 hours/6 months, and TPH Spectra HV (control)(4) 24 hours/6 months. The samples were stored at standard conditions for 24 hours or 6 months. Then dipped in Procyon red solution. The results show that there is no statistically significant difference between the gingival microleakage of the four groups after 24-hour storage and after 6-month storage.

In the study of Haak et al, 46 64 interproximal boxes were considered, restored with four different materials. The materials were used with two different adhesives each: SonicFill (SF) OFL/X, TetricEvoCeram Bulk fill (TEC) OFL/X, x-tra fil (XF) OFL/X, Permise (P) OFL/X. Following storage in distilled water at 37°C for 24 hours or 180 days, an analysis of the copies in epoxy resin was performed with SEM. The results highlight that there is no statistically significant difference between bulk fill composites and control.

In the study of Peutzfeldt et al, 47 78 cavities restored with three different materials: Filtek SupremeXTE(1), Filtek Bulk Fill(2), and SDR(3) were made. The samples were treated by storage in water for 24 hours, then aging with mechanical brushing (SM) and thermocycling (T). Analysis of the SEM epoxy resin copies before and after artificial aging showed that the SDR material has the smallest marginal gap. There is no difference between Filtek Supreme and Filtek Bulk Fill.

In the study by García Marí et al, 48 80 cavities were analyzed. Restored with two different materials: FiltekTM Bulk Fill A2 (group1) and Filtek Supreme XTE A2B (group2). The samples were subjected to thermocycling, then immersed in a 0.5% basic fuchsin solution. The analysis showed that the percentages of the microleakage score show nonstatistically significant differences between the various groups.

## Discussion

A narrative summary of the studies included in the review was made. It was not possible to carry out a meta-analysis due to the heterogeneity of the methods and the evaluation of the results of the individual studies.

Despite the lack of a standardized protocol for the evaluation of bulk fill composites at the root cement interface, the studies analyzed do not reveal statistically significant differences in terms of marginal adaptation between the class of nonbulk fill composites and the class of bulk fill composites in eight of the studies analyzed 40 41 42 43 44 45 46 48 (463 cavities).

However, three studies show statistically significant differences between tested materials:

Poggio et al 39 showed that there is a statistically significant difference in terms of microinfiltration following immersion in basic fuchsin dye: in particular in their study the products SonicFill (bulk) and Grandio (nanohybrid) have better performances compared with GrandioSo (nanohybrid) and SDR (bulk fill), with Filtek Supreme having the worst infiltration.

Peutzfeldt et al 47 instead show that the SDR material has the smallest marginal gap compared with Filtek Supreme (control) and Filtek bulk fill (bulk fill), which do not show statistically significant differences between them.

Juloski 38 et al point out that the SDR material presents the greatest microleakage.

Even in the studies that show a statistically significant difference between the materials tested, it should be emphasized that the best or worst marginal seal performance is not relative to the overall class of bulk composites, but is the prerogative of the individual products.

Technical skill, the need for isolation under the dam, and the time required to carry out the restoration are elements that cannot always be fully satisfied due to factors related to the skill of the operator, the anatomical site of the restoration, and errors that cannot be completely eliminated. To complete a restoration quickly, the industry has developed in the last 10 years resin-based composite materials with peculiar characteristics that allow them to be deposited in incremental layers up to 4 mm while reducing the time required and the possibility of making technical errors. These operational possibilities become particularly useful in conditions such as cavity preparations with root cement margin where we find a tissue that is not ideal for adhesive techniques, exposed to the high risk of infiltration during the isolation phase. The cavity margin of the cement preparation therefore represents a weak point in the interface of the adhesive restoration. 49 50 51 52 53 54 In the presence of these anatomical assumptions, new technologies can be useful providing materials with high adaptability and marginal seal, with low polymerization shrinkage and therefore with reduced tension forces on the adhesive interface. Successfully Lo Giudice et al 8 used a flowable composite at the interface with the root cementum to exploit the low elastic modulus of the material to reduce the effects of polymerization stress; however, it was impossible to obtain a marginal seal. Better standardization of the cavity would be useful; also in reference to the fact that bulk-fill composites seem to be more advantageous especially in deep cavities. 16 55

A great variability is also present in the evaluation phase due to the presence of different techniques and consequently, of different methods for evaluating marginal integrity.

A further confounding factor is that materials with very heterogeneous characteristics are included in the class of bulk fill resin-based composite materials, which are further classified, in a variously incomplete way, based on viscosity.

However, with all the limitations described above, the selected studies show that there is substantially no statistically significant difference in the marginal integrity of the cement margins of cavity preparations restored with bulk fill composites compared with those restored with non-bulk composite.

Our results are in line with previous research: there is a significant heterogeneity within the class of bulk fill composites 55 56 not a better or worse seal is clearly observed. 40 43 52 57 58 59 60

Furthermore, there is no need for the incremental technique that is highly sensitive, operator dependent, and prone to inevitable errors (contamination between the layers, incorporation of air bubbles between the layers) that can compromise the quality of the restoration. 54 61 62

### Limitations

Unfortunately, must be highlighted, an extreme heterogeneity of the studies: even though these are *in vitro* experiments, it is not possible to define the standard dimensions of the cavities of each study, since each investigator has chosen slightly different dimensions.

The extreme variability is found event in the choice of the material. Considering that it is difficult to classify bulk composites on the basis of their chemical characteristics, we carry out a classification on the basis of the macroscopic physical characteristics, which, however, is largely incomplete as they are also minimal variations in the content of filler or monomer 63 or molecules that accompany and modulate the photopolymerization is accompanied by variability of the physical behavior of the material, variability of the elastic modulus are significant even within the same category of materials in consideration of the fact that the filler content, which varies between the different bulk composites strongly influences the elastic modulus of the material. Certainly, a learning curve in the use of these materials is essential and furthermore it is necessary to consider the operator-addiction. The use of magnification systems, for example, as already evaluated in the literature, can be essential in the clinical results of this type of practice. 33 64 65 66

The variability is also reflected in the absence or presence of artificial aging of the restoration and in the method of evaluating the marginal adaptation that, in fact, is performed in different ways in these studies: immersion in 0.5% basic fuchsin solution, immersion in 10% methylene blue, immersion in 1% methylene blue, immersion in 2% Procion Red solution, and SEM analysis of the copies in epoxy resin.

Another fundamental aspect to underline is that the interproximal regions of bulk fill restorations have not always been coated with a traditional composite (as indicated by the manufacturer); this could have worsened the performance of the composites, as it was observed that the superficial microhardness of the two low viscosity bulk fill composites (SDR Dentsply; Venus Bulk Fill, Heraeus Kulzer) is significantly reduced by their exposure to solvents that simulate food solvents, hence the manufacturers' recommendations to cover the material with a cap of material composite. 67 However, it has also been shown that the same SDR has, in a statistically significant manner, a lower polymerization stress than some traditional composites and some flowable composites 35 and also compared with other low viscosity (base) bulk fill composites. 68 69 70

## Conclusions

The bulk fill technique is characterized by a shorter operating time and less dependence on the operator, and fewer procedural errors are detectable.

In conclusion, it can be stated that, with the limitations of the present study, from the data extracted from the review of the literature, there are no clear differences that indicate a better or worse marginal seal with bulk fill composites, while the time savings and operational simplification make it possible to make the direct restoration technique less dependent on the operator's expertise. Further studies like this could further clarify whether the use of these materials is more versatile than expected. Being able to use materials like this, which still demonstrate promising results, can make clinical practice easier and faster.

However, further studies are needed to gather further information, using a shared and standardized protocol that allows comparing the results of the different studies.
